# *AKR1C2* and *AKR1C3* expression in adipose tissue: Association with body fat distribution and regulatory variants

**DOI:** 10.1016/j.mce.2021.111220

**Published:** 2021-05-01

**Authors:** Giada Ostinelli, Jinchu Vijay, Marie-Claude Vohl, Elin Grundberg, Andre Tchernof

**Affiliations:** a Centre de Recherche de l’Institut Universitaire de Cardiologie et Pneumologie de Québec-Université Laval, 2725 Chemin Sainte-Foy, G1V 4G5, Québec City, Québec, Canada; b École de Nutrition, Université Laval, 2425 Rue de l’Agriculture, G1V 0A6, Québec City, Québec, Canada; c Department of Human Genetics, McGill University, Montréal, Quebec, Canada; d Centre Nutrition, Santé et Societé (NUTRISS)-Insitut sur la Nutrition et les Aliments Fonctionnells (INAF), Université Laval, Québec City, Québec, Canada; e Children's Mercy Research Institute, Children's Mercy Kansas City, Kansas City, MO, USA

**Keywords:** *AKR1C2*, Gene expression, Body fat, Single nucleotide polymorphism, Adipose tissue, Androgens

## Abstract

**Background:**

Changes in androgen dynamics within adipose tissue have been proposed as modulators of body fat accumulation. In this context, *AKR1C2* likely plays a significant role by inactivating 5α-dihydrotestosterone.

**Aim:**

To characterize *AKR1C2* expression patterns across adipose depots and cell populations and to provide insight into the link with body fat distribution and genetic regulation.

**Methods:**

We used RNA sequencing data from severely obese patients to assess patterns of *AKR1C2* and *AKR1C3* expression in abdominal adipose tissue depots and cell fractions. We additionally used data from 856 women to assess *AKR1C2* heritability and to link its expression in adipose tissue with body fat distribution. Further, we used public resources to study *AKR1C2* genetic regulation as well as reference epigenome data for regulatory element profiling and functional interpretation of genetic data.

**Results:**

We found that mature adipocytes and adipocyte-committed adipocyte progenitor cells (APCs) had enriched expression of *AKR1C2*. We found adipose tissue *AKR1C2* and *AKR1C3* expression to be significantly and positively associated with percentage trunk fat mass in women. We identified strong genetic regulation of *AKR1C2* by rs28571848 and rs34477787 located on the binding sites of two nuclear transcription factors, namely retinoid acid-related orphan receptor alpha and the glucocorticoid receptor.

**Conclusion:**

We confirm the link between *AKR1C2,* adipogenic differentiation and adipose tissue distribution. We provide insight into genetic regulation of *AKR1C2* by identifying regulatory variants mapping to binding sites for the glucocorticoid receptor and retinoid acid-related orphan receptor alpha which may in part mediate the effect of *AKR1C2* expression on body fat distribution.

## Highlights

• *AKR1C2* is highly expressed in human mature adipocytes and adipocyte-committed adipocyte progenitor cells (APCs).
• *AKR1C2* expression in subcutaneous adipose tissue is positively associated with trunk body fat mass in women.
• The retinoid acid-related orphan receptor alpha (RORA) and the glucocorticoid receptor (GR) are likely to be important regulators of *AKR1C2* expression in adipose tissue.

## 1 Introduction

Obesity is associated with numerous cardiometabolic complications including the metabolic syndrome (Shungin et al., 2015), type 2 diabetes (T2D) and cardiovascular diseases (CVD). Despite this widely acknowledged association, not all individuals with a body mass index (BMI) above 30 kg/m^2^ develop metabolic impairments (Tchernof and Despres, 2013). In this framework, body fat distribution is an established factor strongly contributing to a deleterious cardiometabolic profile (Tchernof and Despres, 2013). Indeed, increased abdominal obesity, linked to ectopic fat and in particular excess visceral adipose tissue (VAT), positively correlates with insulin resistance and atherogenic dyslipidemia (Tchernof and Despres, 2013). More so than increases in subcutaneous adipose tissue (SAT) mass, VAT expansion is associated with adipose tissue dysfunction, a set of histologic and cellular characteristics including, among others, adipocyte hypertrophy (Michaud et al., 2016; Laforest et al., 2015), impaired adipogenesis (Lessard et al., 2014; Gustafson et al., 2019), increased macrophage infiltration (Michaud et al., 2012), fibrosis (Esteve et al., 2019; Michaud et al., 2005), insulin resistance (Michaud et al., 2016; Laforest et al., 2015), decreased lipid uptake (Normand-Lauziere et al., 2010) and impaired triglyceride synthesis (Cote et al., 2014). The protective role of SAT has been reported in a number of studies (Lessard et al., 2014; Esteve et al., 2019). The underlying hypothesis is that SAT may be more prone to adipogenesis than VAT, thereby providing better expansion capacity and lipid handling in the post-prandial phase (Neeland et al., 2019). The strong association between excess VAT accumulation, adipose tissue dysfunction and systemic metabolic imbalance (Neeland et al., 2019) additionally stresses the importance of body fat distribution.

Body fat distribution is a trait showing strong heritability, in some cases as high as 50% (Tchernof and Despres, 2013). Regarding possible DNA variants that may be inherited, single nucleotide polymorphisms (SNPs) are certainly one of the most frequent point-modifications involved. Since the advent of high-throughput technologies allowing whole-genome analysis, a number of genome-wide association studies looking into SNPs related to body fat distribution have been published (Shungin et al., 2015; Rask-Andersen et al., 2019; Fehlert et al., 2017; Chu et al., 2017; Glastonbury et al., 2019; Benton et al., 2013; Randall et al., 2013). Some found a considerable number of SNPs that were related to markers of adipose tissue function, including lipid accumulation (Shungin et al., 2015; Benton et al., 2013), adipocyte differentiation (Chu et al., 2017) or glycemic control (Shungin et al., 2015). Most of these studies reported sex-specific effects (Shungin et al., 2015; Rask-Andersen et al., 2019; Fehlert et al., 2017; Chu et al., 2017; Randall et al., 2013), suggesting a possible involvement of sex hormones. Consistent with these findings, androgens such as testosterone (T) and 5α-dihydrotestosterone (5α-DHT) have been identified as modulators of body fat distribution in both men and women (Tchernof et al., 2018). A comprehensive review of available literature showed that women with high serum T display android obesity whereas low T is related to more abdominal fat accumulation in men (Tchernof et al., 2018). From the molecular point of view, aldo-ketoreductases 1C such as AKR1C2 and AKR1C3 may be of importance for local androgen metabolism because they can locally modulate 5α-DHT and T levels (Tchernof et al., 2015). AKR1C3 possesses a significant 17β-hydroxysteroid dehydrogenase (17β-HSD) activity (Zhang et al., 2000), for example reducing the precursor androstenedione to T (Tchernof et al., 2015). AKR1C2 has 3α-HSD activity and is responsible for the specific inactivation of the potent androgen 5α-DHT into 5α-androstane-3α,17β-diol (Zhang et al., 2000; Blouin et al., 2003), a much weaker ligand of the androgen receptor (AR). Specific downregulation of AKR1C2 in preadipocytes was found to potentiate the inhibitory effect of 5α-DHT on adipogenic differentiation (Veilleux et al., 2012). Thus, the adipose-specific expression of enzymes involved in androgen metabolism are linked with obesity phenotypes and may be further involved in determining the patterns of body fat distribution in humans.

In the present study, we aimed at characterizing the genetic variants of *AKR1C2* and to further relate its expression in adipose tissue cell populations and body fat distribution. We hypothesized that increased *AKR1C2* expression is associated with abdominal obesity and that analysis of genetic variants in key regulatory elements suggests putative mechanisms related to possible hormonal control of *AKR1C2* expression.

## 2 Methods

### 2.1 MuTHER cohort

We used data generated from the Multiple Tissue Human Expression Resource (MuTHER) study that includes 856 European descent females taking part in the TwinsUK Adult twin registry (Grundberg et al., 2012). The study received ethical approval and written informed consent was signed from participants before sample collection. Trunk fat mass was measured by dual-energy X-ray absorptiometry (DXA) according to the manufacturer protocol (Glastonbury et al., 2016). Subcutaneous adipose tissue (SAT) samples were obtained under the umbilicus (8 mm deep), fat was weighted and immediately frozen in liquid nitrogen. The detailed methodology of SAT processing has already been published (Grundberg et al., 2012). Briefly, tissues were homogenized and RNA extracted using TRIzol Reagent (Invitrogen). RNA expression, in replicates, was measured by Illumina Human HT-12 V3 BeadChips (Illumina). Illumina probe annotation was manually analyzed using NCBI Build 36 genome and only probes with no mismatches and already identified on Ensembl or RefSeq were kept for analysis. In addition, for the purposes of the current study, the probes were linked to transcript variants and DNA locations using ensembl GrCh38 Biomart (http://uswest.ensembl.org/biomart).

Expression-phenotype associations were performed as described previously (Small et al., 2011) using a linear mixed-effects model, implemented with the *lme4* R package. The model was adjusted for both fixed effects (age, batch) and random effects (family relationship and zygosity).

As described previously (Grundberg et al., 2012), by taking advantage of the twin design it is possible to estimate the genetic and environmental contribution of gene expression heritability. Briefly, the classical ACE model was applied, which allows the segregation of genetic expression variance into Additive genetic (A), Common environment (C) and unique Environment (E) among mono- and dizygotic twins.

### 2.2 Single-cell sequencing cohort

We used recently published data on stroma-vascular fraction (SVF) single-cell RNA sequencing to examine AKR1C2 expression in various cell types of adipose tissue (Vijay et al., 2020). 14 patients (10 women and 4 men) awaiting for bariatric surgery were recruited through the Biobank of the Quebec Heart and Lung Institute (IUCPQ-Université Laval). Of these, 5 were diagnosed with T2D (2 men and 3 women). However, due to the small sample size, analyses based on T2D diagnosis were not performed. Adipose tissues from the greater omentum (VAT) and abdominal subcutaneous adipose compartment (SAT) was taken at the time of the surgery. This study was approved by the institutional ethics committee and signed consent was obtained from all participants.

The complete methodology used can be found in (Vijay et al., 2020). Following isolation of the SVF, cells were thawed, diluted in DMEM/F12 (10% fetal bovine serum and 1% penicillin-streptomycin) and centrifugated. The pellet was then resuspended in supplemented DMEM/F12 and 12,000 cells per patient were loaded onto the Chromium Single Cell A Chip (10x Genomics). Cell capture, cDNA amplification and library preparation were performed using Chromium Single Cell 3’ Library and Gel Bead Kit v2 (10x Genomics). Following the evaluation of both library size and concentration, samples were sequenced using Illumina HiSeq (Vijay et al., 2020). For the primary analysis, we used Cell Ranger v.2.1.0 software from 10XGenomics with GRCh38 as reference. The R package *Seurat* (Butler et al., 2018) was used for secondary analysis such as filtering and dimensionality reduction.

The SAT progenitor cell population was divided into 5 clusters, according to gene expression signature. SAT progenitor population 1 (SP1) and SP3 expressed typical adipocyte progenitor cell (APC) markers such as *MGP, ADOP, CXCL14*. In the original study (Vijay et al., 2020), the two populations were considered as separate because only SP1 abundance positively correlated with plasma glucose levels and was more prevalent in patients with T2D. SP2 was associated to adipogenic commitment (*APOE*, *FABP4*, *CEBPB* and *CD36* expression). On the other hand, SP4 displayed a fibroblast-like signature (*COL1A1*, *COL6A3* and *COL6A1*) while SP5 showed inflammatory gene expression and related to hematopoietic stem cells progenitors (*CCL5*, *IL7R*, *IL32* expression) (Vijay et al., 2020). Differential expression between clusters was evaluated by the Wilconxon rank sum test in *Seurat*.

### 2.3 Adipose tissue RNA-sequencing cohort

We included SAT and VAT samples from 43 individuals with severe obesity (BMI ≥35.5 kg/m^2^, age 29–65 years) awaiting bariatric surgery and recruited through the Biobank of the Quebec Heart and Lung Institute (IUCPQ-Université Laval). Whole adipose tissue was collagenase-digested using a modified version of the Robdell method within 30 min of collection. The isolated mature adipocytes, SVF and whole adipose tissue samples were cryopreserved and stored in liquid nitrogen. From the frozen samples, 0.5 to 3 millions cells were resuspended in 500 μL TRIzol Reagent. RNA was extracted using the miRNeasy Mini Kit (Qiagen) according to the manufacturer's protocol. RNA library preparations were carried out on 500 ng RNA with RNA integrity number (RIN) > 7 using Illumina TruSeq Stranded Total RNA Sample preparation kit. Final libraries were analyzed on a Bioanalyzer and sequenced on the Illumina HiSeq 2500. The sequence reads were 100–125 bp in length and pair-ended.

After trimming for quality (phred33 ≥ 30) and length (n ≥ 32), Illumina adapters were clipped off using Trimmomatic v.0.35 (Bolger et al., 2014). Filtered reads were then aligned to the GRCh37 human reference wing STAR v.2.5.3a (Dobin et al., 2013). Raw read gene counts were obtained using htseq-count v.0.6.0 (Anders et al., 2015). Differential gene expression analysis was done using DeSeq2 v.1.18 (Love et al., 2014).

The same samples were used to assess chromatin state in human mature adipocytes using transposase-accessible chromatin using sequencing (ATAC-Seq) (Buenrostro et al., 2013).

### 2.4 Genome mapping

Data on AKR1C2 gene were retrieved from publicly available databases such as NCBI (tissue expression in humans) and the Genotype-Tissue Expression (GTEx) resource (https://gtexportal.org/) (mRNA variant expression by tissue).

### 2.5 Chromatin modifications

Variants in linkage disequilibrium (LD) with the sentinel SNP rs28571848 were retrieved from LDlink (https://ldlink.nci.nih.gov/?tab=home) using genome version GRCh37. Variants with R^2^ values > 0.8 were considered to be in high LD and were further analyzed. Chromatin modification, accessibility and transcription factor (TF) binding tracks were obtained by searching the ENCODE project database (https://www.encodeproject.org/) for experiments associated with mature or differentiated adipocytes (where possible) and mapped to GRCh38 genome version. Data on chromatin modification such as the mono- and trimethylations of lysin 4 on histone 3 (H3K4me1 and H3K4me3) and the acetylation of lysin 9 on histone 3 (H3K9ac) were downloaded from the project ROADMAP epigenomics. Glucocorticoid-receptor (GR) binding (coded by the NR3C1) and histone acetyltransferase E1A-associated protein p300 (EP300) were retrieved from Chip-seq analysis conducted in the A594 cell line stimulated with dexamethasone for 8 and 7 h respectively. Finally, gene loci and variant mapping were downloaded from NCBI RefSeq Gene and SNPdb. Data were plotted with the help of Integrative genomics viewer (IGV) (Robinson et al., 2017).

### 2.6 Expression quantitative trait loci

The publicly available 8th version of the GTEx resource (https://gtexportal.org/), was used to collect data of SNP effects on AKR1C2 and AKR1C3 expression in adipose tissue. GTEx is a well-known resource providing a rich catalog of densely profiled tissue samples including subcutaneous, visceral and mammary adipose tissues (SAT, VAT and MAT) with precomputed association analysis of genotypes and expression to identify quantitative trait loci (eQTL) (The GTEx Consortium atlas, 2020). Data mining was used on open-source data to isolate the information on *AKR1C2* and *AKR1C3* expression in human adipose tissues.

### 2.7 Variants prioritization

We prioritized variants in high LD using scoring algorithms such as RegulomeDB (Boyle et al., 2012; Dong and Boyle, 2019) and IW scoring system (Wang et al., 2018). The first is an online database combining data from the ENCODE project, manual annotations and algorithms to predict the effect of a single-base mutation on chromatin accessibility and TF binding (Boyle et al., 2012). Just like RegulomeDB, a number of similar algorithms have been published and made publicly available. IW scoring system pools together a number of published algorithms with experimental chromatin data available on ENCODE and Ensembl. The output gives an overall score helping the ranking of genetic variants. To prioritize SNPs, we used IW score (K10), which combines data from CADD score, DeepSEA, EIGEN, FATHMM, FunSeq2, GWAVA, ReMM, ENCODE, Ensembl and FANTOM5 (Wang et al., 2018).

Scores were normalized in R, reversing Regulome and DeepSEA scales and standardizing each score's vertical height by setting minimum to zero and maximum to one. Each score was then centered using its median.

## 3 Results

### 3.1 Characterization of AKR1C2 expression across adipose tissue types

**Fig. 1:**
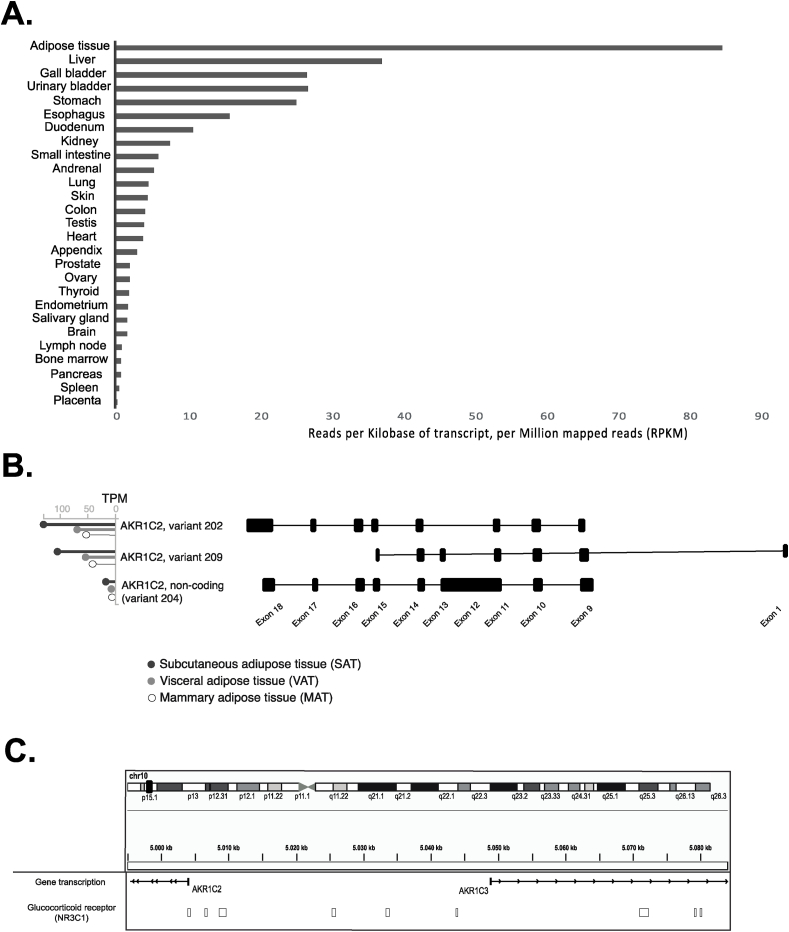
**AKR1C2 expression.** AKR1C2 expression in human tissues (A), most common transcripts of AKR1C2 in adipose tissue (B, modified from GTEx), AKR1C2 locus on chromosome 10 (C, modified from IgV).

Tissue-specific *AKR1C2* expression was gathered from the National Center for Biotechnology Information (NCBI) database, which includes summary statistics regarding tissue-specific expression of protein-coding genes in humans (Fagerberg et al., 2014). We found *AKR1C2* to have its highest expression in adipose tissue with levels up to 84.8 reads per kilobase (kb) of transcript, per million of reads (RPKM). Its expression in adipose tissue was more than double that observed in the liver (Fig. 1a). We then accessed the GTEx resource which contains genome-wide expression data on 54 tissue types across 948 donors (The GTEx Consortium atlas, 2020). As expected, GTEx reported that among adipose tissue types, SAT had the highest *AKR1C2* expression, where AKR1C2-202 (ENST00000421196.7) was the most abundant transcript (Fig. 1b). Interestingly, we also noted that an alternative non-coding *AKR1C2* transcript is expressed in adipose tissue, although its expression was significantly lower than the one observed for the coding transcripts. The *AKR1C2* maps to the reverse strand of chromosome 10 (Chr10:5060223), close to *AKR1C3*. Indeed, as for genome version GRCh37, the two genes were separated by only 17.3 kb. As shown in Fig. 1c, as many as nine glucocorticoid responsive elements (GRE) were found upstream of *AKR1C2*.

### 3.2 Patterns of AKR1C2 expression in adipose tissue-derived cell populations

Firstly, we compared expression pattern in the two main cell fractions found in adipose tissue, including adipocytes and the SVF using data from the adipose tissue RNA-sequencing cohort. We found that both in SAT and VAT, adipocytes have a more abundant *AKR1C2* expression than SVF (log2 fold change: 4.60, adjusted p-value: 9.55 E^−125^, log2 fold change: 5.85, adjusted p-value: 1.25 E^−161^ for SAT and VAT, respectively). A similar pattern, but less pronounced, was found for *AKR1C3* expression across cell fractions (log2 fold change: 3.98, adjusted p-value: 2.53 E^−85^, log2fold change: 4.57, adjusted p-value: 1.1 E^−76^ for SAT and VAT, respectively).

**Fig. 2:**
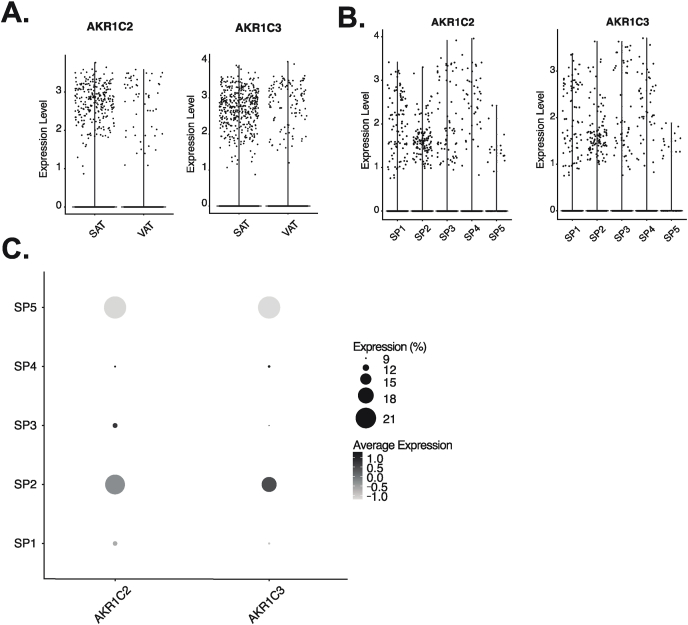
**Characterization of *AKR1C2* and *AKR1C3* expression in the stroma vascular fraction of adipose tissue at the single-cell level**. *AKR1C2* and *AKR1C3* expression in subcutaneous (SAT) and visceral (VAT), all cells combined. Expression (mean ± SD): *AKR1C2* SAT: 2.73 ± 0.48; VAT: 2.67 ± 0.72; *AKR1C3* SAT: 2.71 ± 0.52; VAT: 2.86 ± 0.60 (A); in subcutaneous clusters of the stroma vascular fraction (B), SP1and SP3: adipocyte progenitor cells (APCs), mean ± SD expression SP1: *AKR1C2* 2.02 ± 0.69; *AKR1C3* 1.96 ± 0.76; SP3: *AKR1C2* 1.46 ± 0.39; *AKR1C3* 2.26 ± 0.70), SP2: adipocyte-committed APCs (mean *AKR1C2* expression 2.09 ± 0.85; mean *AKR1C3* expression 2.1 ± 0.89), SP4: fibroblasts (mean *AKR1C2* expression 2.5 ± 0.69; mean *AKR1C3* expression 2.25 ± 0.70), SP5: hematopoietic stem cell progenitors (mean *AKR1C2* expression 1.66 ± 0.4; mean *AKR1C3* expression 1.70 ± 0.53); percent of cells expressing and average expression of *AKR1C2* and *AKR1C3* in the different subcutaneous clusters (C).

Next, we used single-cell RNA sequencing data of the SVF derived from 14 patients undergoing bariatric surgery (Vijay et al., 2020) and compared *AKR1C2* expression across APC populations in both SAT and VAT. Similarly to what was hinted by GTEx, we confirmed that *AKR1C2* was significantly more expressed in SAT than VAT (log fold change: 0.20, adjusted p-value: 3.74 E^−^^24^, Fig. 2a). Given the higher expression of *AKR1C2* in SAT, we focused on SAT progenitor cells only. When combining men and women together, we found *AKR1C2* to be expressed more abundantly in adipocyte-committed APCs expressing *APOE*, *FABP4* and *CEBPB* (labeled SP2 cells) compared to the other populations such as SP1 and SP3 (expressing APCs markers), SP4 (associated with extracellular matrix synthesis and fibrosis) or SP5 (representing hematopoietic stem cell progenitors) (Fig. 2b and c). Indeed, SP2 expressed significantly higher *AKR1C2* compared to SP1,3 and 4 combined (log fold change: 0.05, adjusted p-value: 2.74 E^−05^).

### 3.3 Linking AKR1C2 expression to phenotype

Using the MuTHER adipose tissue resource, which combines SAT gene expression in 856 deeply phenotyped women, we investigated the association between *AKR1C2* and trunk fat mass. We found *AKR1C2* expression to be positively and significantly associated with percent trunk fat mass, even after adjustment for BMI (Table 1).

**Table 1:** **Subcutaneous adipose tissue gene expression in the Multiple Tissue Human Expression Resource** (MuTHER, part of TwinsUK). Associations with percentage trunk fat mass or trunk fat mass adjusted for BMI and the expression of AKR1C2 and AKR1C3 in subcutaneous adipose tissue.

Probe ID	Location	Gene	Trunk fat mass (%)			Trunk fat mass (%), BMI adjusted		
			P-value (ANOVA)	Q value	Beta	p-value (chi square)	Q value	Beta
ILMN_2412336	chr10:5011031:5022080	AKR1C2	2.2 E^–^^49^	2.5 E^–^^47^	0.023	7.8 E^–^^30^	3.3 E^–^^27^	0.026
ILMN_1713124	chr10:5139765:5139814	AKR1C3	2.1 E^–70^	8.7 E^–68^	0.037	4.3 E^–^^31^	2.1 E^–^^28^	0.034

We also found this association for *AKR1C3* expression. Interestingly, the probe having the strongest association with percent trunk fat mass in women (BMI-adjusted p-value: 7.8 E^−30^) was tracked to the AKR1C2-202 transcript variant (ENST00000421196.7) which we described above being the most prevalent transcript in adipose tissue (Fig. 1b). However, we found an association for the *AKR1C2* transcript variant 209 (ENST00000604507.5), although less significant (BMI-adjusted p-value of 5.2 E^−05^).

### 3.4 Genetic regulation of AKR1C2 expression

We sought to estimate the regulation of *AKR1C2* expression and again used the MuTHER cohort to assess the genetic and non-genetic contribution to *AKR1C2* expression variation in adipose tissue specifically. As we have shown before (Grundberg et al., 2012), MuTHER includes dizygotic and monozygotic twins and allows for the estimation of heritability (h^2^) of expression levels as well as shared and unique environmental contributions. Results point to a heritability of *AKR1C2* expression of approximately 40%, while the environment seems to contribute to a larger extent in adipose tissue with an h^2^ of 0.56.

We then aimed to map the underlying common genetic effect of *AKR1C2* expression and used the GTEx database for genotype-expression association. Our analyses identified rs28571848 with a minor allele frequency smaller than 20% (Table 2) as the strongest association with SAT expression level of *AKR1C2 (*p = 7.8E^−^^12^). Interestingly, the same variant (rs28571848) was also the top associated SNP with SAT *AKR1C3* expression (p = 2.1E^−^^33^).

**Table 2:** **Minor allele frequency in the 1000 genome and TwinsUK cohorts.** The two alleles found on the forward DNA strand (most frequent/rare) are listed, followed by the minor allele frequency (MAF) reported by 1000 genome (1000G) and TwinsUK. Data were retrieved from RefSNP.

SNP	Allele	Minor allele frequency (1000G)	Minor allele frequency (TwinsUK)
rs28571848	C/T	15.3%	18.6%
rs12411321	T/C	12.5%	17.6%
rs34477787	C/A	12.4%	17.7%
rs1937907	G/A	16.9%	–

**Fig. 3:**
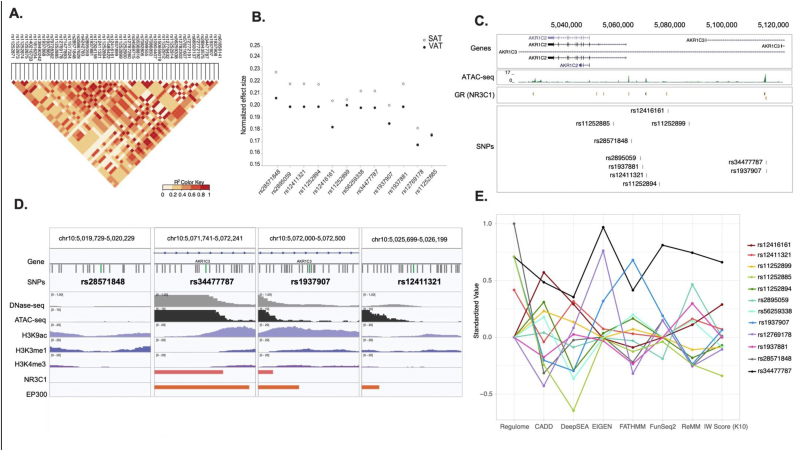
**Identification of a possible causal single nucleotide polymorphism**. Single nucleotide polymorphisms in linkage disequilibrium with rs28571848 (A), effect on AKR1C2 and AKR1C3 gene expression in both subcutaneous and visceral adipose tissue of possible causal single nucleotide polymorphisms (B), chromatin state obtained with ATACseq of mature adipocytes upstream AKR1C2 and localization of common genetic variants (C), epigenetic environment around rs28571848, rs34477787, rs1937907, rs12411321 (D, modified from IgV), plot pooling the results obtained with Regulome, CADD score, DeepSEA, EIGEN, FATHMAN, FunSeq2, RaMM and IWScore (K10) scoring algorithms (E).

However, rs28571848 was in strong linkage disequilibrium (LD) with a number of other SNPs (Fig. 3a). Indeed, some SNPs having R^2^ values ≥ 0.8 were localized more than 20 kb from the *AKR1C2* transcription start site. We found that all of the SNPs in LD with rs28571848 have the same direction of effect for both *AKR1C2* and *AKR1C3* expression, but the most pronounced effects are on *AKR1C3* expression. For both genes, these effects were consistently greater in SAT than in VAT (Fig. 3b). We identified 4 SNPs with a less pronounced influence on AKR1C2 expression: rs12416161, rs1937907, rs12769178 and rs11252885 (Fig. 3b). Using additional data on open chromatin state gathered from our adipose tissue RNA-sequencing cohort (see method section) using ATAC-seq on SAT-derived human mature adipocytes (Fig. 3c), we found that rs34477787, rs28571848 and rs12411321 overlapped open chromatin in human mature adipocytes. On the other hand, data from the Roadmap epigenomic project revealed that rs11252894, rs193788, rs12769178, rs11252885 are found in heterochromatin or a quiescent state (data not shown).

In light of the data collected, we decided to focus our efforts on rs28571848, rs34477787, rs12411321 and rs11252899. To better understand the impact that these SNPs may have on adipose tissue gene expression and their likelihood to affect chromatin state, we interrogated the Regulome database (Regulome DB). We found that a number of variants were in close proximity with many transcription factor binding sequences, identified by either chromatin immunoprecipitation (Baars et al., 2016), position weight matrixes (PWM) or motif. In particular, we found that rs28571848 was within the retinoic acid-related orphan receptor alpha (RORA) sequence, that rs12411321 was associated with the subunit 2A of polymerase II during dexamethasone stimulation, and that rs11252899 was in the sequence recognized by Oct-1 (data not shown). rs34477787 was found in the sequence recognized by GR, while rs1937907 was located on a sequence that co-immunoprecipitated with EP300 (Fig. 3c and d). In light of these results, we decided to further investigate the chromatin state around the four likely functional SNPs, being rs28571848, rs12411321, rs34477787 and rs1937907. Overall, data collected from ENCODE show that rs34477787, rs1937907 and rs28571848 may be located in an accessible locus with open chromatin (Fig. 3d).

To better understand the impact of these SNPs, we pooled together the results gathered using both Regulome DB and IW scoring system. As illustrated in Fig. 3e, our results clearly identified rs34477787 as the SNP that is more likely to affect both chromatin state and gene transcription. Indeed, among the 10 SNPs tested no other SNP consistently performed as well as rs34477787 across the algorithms tested. Interestingly, although rs34477787 outperformed the other SNPs, according to Regulome DB, rs28571848 is more likely to be a regulatory variant (rank: 2b, 3a probability: 0.75, 0.78 for rs28571848 and rs34477787 respectively).

## 4 Discussion

Our aim was to relate *AKR1C2* expression in adipose tissue to body fat distribution and to characterize *AKR1C2* upstream variants known to increase its transcription. We found *AKR1C2* to be strongly expressed in adipose tissue, where the transcript variant 202 (ENST00000421196.7) was the most expressed. Our analyses of adipose cell populations confirmed the upregulation of *AKR1C2* expression in mature adipocytes of both SAT and VAT. We then investigated the stromal fraction of adipose tissue and identified a specific cell population cluster upregulating *AKR1C2*. Additional analyses on gene expression in these cells revealed that this population belonged to the adipocyte committed APCs in SAT. The increase of *AKR1C2* expression as a function of adipogenic commitment and more generally, preadipocyte differentiation, are supported by the literature, where *AKR1C2* has been found to be one of the most expressed steroid-converting enzymes in mature adipocytes (Veilleux et al., 2012). AKR1C2 plays an essential role in androgen turnover in adipose tissue (Zhang et al., 2000). Indeed, by inactivating 5α-DHT during the early steps of adipogenesis, AKR1C2 may facilitate lipid accumulation into the differentiating pre-adipocytes (Veilleux et al., 2012).

Our results show a strong association between *AKR1C2* as well as *AKR1C3* expression and percent trunk fat mass in women, where the most compelling evidence came from the association with *AKR1C2* transcript variant 202. A number of studies indicated that the intra-adipose expression of *AKR1C2* and *AKR1C3* expression in adipose tissue is positively associated with adiposity indices. In our previously published works, we reported a significant positive association between *AKR1C2* expression as well as its enzymatic activity in adipose tissue and visceral fat accumulation (Blouin et al., 2003). The same association was found between adipocyte cell size and *AKR1C2* expression or activity in adipose tissue. Similar results were found in a second sample of 45 participants, were *AKR1C2* and *AKR1C3* expression in adipose tissue were positively correlated with the waist-hip ratio (Wake et al., 2007), another marker of preferential android fat accumulation. Recently, another group suggested a possible role of both steroid-converting enzymes in the pathogenesis of human obesity. Irrespective of sex, in BMI-discordant twins, intra-adipose expression of *AKR1C2* and *AKR1C3* were found to be higher in the heavier twin (Vihma et al., 2017, 2018). As a consequence, serum concentration of 5α-DHT were found to differ, with the heavier twin having the lower concentration (Vihma et al., 2017, 2018). Interestingly, the difference in serum concentration between the leaner and the heavier co-twin was inversely correlated with their difference in both abdominal and subcutaneous fat accumulation (Vihma et al., 2017, 2018). In addition, AKR1C3 has been recently shown to have as preferred substrate androgen precursor 11- ketoandrostenedione (11KA4) (Barnard et al., 2018). 11-hydroxy and 11-ketoandrogens have been shown to activate the androgen receptor to a greater extent as opposed to classical androgens (Pretorius et al., 2016) and to be associated to a number of pathological conditions such as the polycystic ovary syndrome (O'Reilly et al., 2017; Turcu et al., 2020). Hence our results may raise interesting questions on this developing field of research. Our analysis further supports the role of *AKR1C2* in adipose tissue accumulation and body fat distribution and opens new opportunities for research on 11-hydroxy and 11-ketoandrogens.

We additionally searched for possible genetic variation that might increase *AKR1C2* expression in adipose tissue and narrowed down to two SNPs: rs28571848, rs34477787, while excluding rs1937907. Indeed rs1937907, associated with the histone acetyltransferase EP300, is a known coactivator of GR (Kino et al., 1999). The enzyme has been found to bind simultaneously to the transcription machinery and to the GR itself (Kino et al., 1999). This, added to the fact that rs34477787 and rs1937907 were very closely located, led us to the assumption that rs34477787 rather than rs1937907, was more likely to have a functional effect. Regarding rs28571848, this SNP is linked to RORA, a nuclear receptor that has been found to participate in pre-adipocyte commitment (Duez et al., 2009), white adipose tissue inflammation in mice (Hams et al., 2020) and metabolic impairments in humans with the metabolic syndrome (Vieira et al., 2014). On the other hand, rs34477787 is found on the locus recognized by the GR. Glucocorticoids are known to induce *AKR1C2* expression in human pre-adipocytes (Veilleux et al., 2012), and the degree of pre-adipocyte sensitivity to induce androgen inactivation following glucocorticoid exposure is positively associated with BMI in humans (Veilleux et al., 2012). The identification of a SNP affecting *AKR1C2* expression and located on a sequence recognized by the GR highlights once more the tight connection that exists between androgen inactivation and glucocorticoids. Finally, our analyses revealed a strong hormonal component likely governing *AKR1C2* expression in adipose tissue. This may explain why this enzyme is rarely identified as a strong contributor to body fat distribution in GWAS studies (Shungin et al., 2015; Rask-Andersen et al., 2019; Fehlert et al., 2017; Chu et al., 2017; Glastonbury et al., 2019; Benton et al., 2013; Randall et al., 2013). Although reasons underlying the less important heritability of *AKR1C2* expression compared to environmental regulation are only speculative at this time, we suggest that the large environmental influences might be associated with the local steroidogenic environment prevailing in adipose tissue in any given situation. Indeed, in light of the known genetic control of *AKR1C2* transcription by other steroids, it is plausible to hypothesize that the load in both androgens and glucocorticoids in adipose tissue can affect *AKR1C2* transcription in humans.

## 5 Conclusion

In conclusion, our study confirms the link between *AKR1C2*, adipogenic commitment and adipose tissue distribution. Moreover, we identified a SNP liking *AKR1C2* gene expression in adipose tissue to glucocorticoid response in humans, consistent with the interplay between glucocorticoids and 5α-DHT inactivation. Additional hormone-mediated regulatory pathways controlling *AKR1C2* transcription and including the retinoic acid receptor, may be involved.

## Funding sources

GO is the recipient of a doctoral scholarship from *Fonds de recherche du Québec-Santé*. EG holds the Roberta D. Harding & William F. Bradley, Jr. Endowed Chair in Genomic Research. AT obtained consulting fees form Bausch Health and research funding from Johnson & Johnson Medical Companies as well as Medtronic for studies unrelated to this manuscript. This work was supported by a 10.13039/501100000024Canadian Institutes of Health Research (CIHR) team grant awarded to E.G and A.T (EGM141898), a CIHR Foundation grant awarded to EG, CIHR grant PJT-169083 to A.T. and the Foundation of the Quebec Heart and Lung Institute, Laval University. The MuTHER Study was funded by a program grant from the 10.13039/100010269Wellcome Trust (081917/Z/07/Z) and core funding for the Wellcome Trust Centre for Human Genetics (090,532). The TwinsUK study was funded by the Wellcome Trust and European Community's Seventh Framework Programme (FP7/2007–2013). The TwinsUK study also receives support from the National Institute for Health Research (NIHR)- funded BioResource, Clinical Research Facility and Biomedical Research Centre based at Guy's and St Thomas' NHS Foundation Trust in partnership with King's College London. The Genotype-Tissue Expression (GTEx) Project was supported by the Common Fund of the 10.13039/100000052Office of the Director of the National Institutes of Health, and by 10.13039/100000054NCI, 10.13039/100000051NHGRI, 10.13039/100000050NHLBI, 10.13039/100000026NIDA, 10.13039/100000025NIMH, and 10.13039/100000065NINDS.
